# 2-[(3,5-Dimethyl-1-phenyl-1*H*-pyrazol-4-yl)methyl­idene]indan-1,3-dione

**DOI:** 10.1107/S1600536811049488

**Published:** 2011-11-25

**Authors:** Abdullah M. Asiri, Abdulrahman O. Al-Youbi, Salman A. Khan, M. Nawaz Tahir

**Affiliations:** a Department of Chemistry, Faculty of Science, King Abdulaziz University, Jeddah 21589, PO Box 80203, Saudi Arabia; b The Center of Excellence for Advanced Materials Research, King Abdulaziz University, Jeddah 21589, PO Box 80203, Saudi Arabia; c University of Sargodha, Department of Physics, Sargodha, Pakistan

## Abstract

In the title compound, C_21_H_16_N_2_O_2_, the five-membered heterocyclic ring makes a dihedral angle of 47.06 (6)° with the attached benzene ring, whereas the indan-1,3-dione ring system and the benzene ring are oriented at a dihedral angle of 21.92 (7)°. In the crystal, inversion dimers linked by pairs of C—H⋯O hydrogen bonds generate *R*_2_^2^(22) loops. Aromatic π–π stacking inter­actions [centroid–centroid distances = 3.8325 (12)–3.8600 (12) Å] also occur.

## Related literature

For background to donor–acceptor chromophores, see: Asiri *et al.* (2006 ▶); Asiri & Khan (2009 ▶); Koyuncu *et al.* (2010 ▶); Kulhanek *et al.* (2011 ▶); Wang *et al.* (2011 ▶). For related structures, see: Belyakov *et al.* (2008 ▶); Fun *et al.* (2010 ▶). For graph-set notation, see: Bernstein *et al.* (1995 ▶).
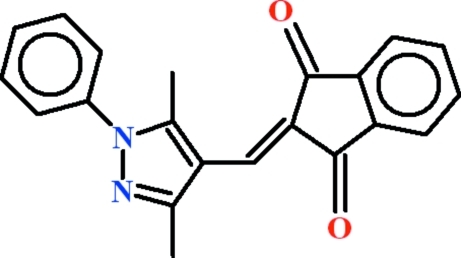


## Experimental

### 

#### Crystal data

C_21_H_16_N_2_O_2_
*M* *_r_* = 328.36
Monoclinic, 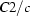

*a* = 14.6655 (3) Å
*b* = 7.8902 (2) Å
*c* = 28.6651 (7) Å
β = 98.251 (1)°
*V* = 3282.61 (13) Å^3^
*Z* = 8
Mo *K*α radiation
μ = 0.09 mm^−1^
*T* = 296 K
0.26 × 0.23 × 0.21 mm

#### Data collection

Bruker Kappa APEXII CCD diffractometer
Absorption correction: multi-scan (*SADABS*; Bruker, 2005 ▶) *T*_min_ = 0.975, *T*_max_ = 0.985
12302 measured reflections
2970 independent reflections
2106 reflections with *I* > 2σ(*I*)
*R*_int_ = 0.034

#### Refinement

*R*[*F* ^2^ > 2σ(*F* ^2^)] = 0.042
*wR*(*F* ^2^) = 0.112
*S* = 1.01
2970 reflections
228 parameters
H-atom parameters constrained
Δρ_max_ = 0.12 e Å^−3^
Δρ_min_ = −0.23 e Å^−3^

### 

Data collection: *APEX2* (Bruker, 2009 ▶); cell refinement: *SAINT* (Bruker, 2009 ▶); data reduction: *SAINT*; program(s) used to solve structure: *SHELXS97* (Sheldrick, 2008 ▶); program(s) used to refine structure: *SHELXL97* (Sheldrick, 2008 ▶); molecular graphics: *ORTEP-3 for Windows* (Farrugia, 1997 ▶) and *PLATON* (Spek, 2009 ▶); software used to prepare material for publication: *WinGX* (Farrugia, 1999 ▶) and *PLATON*.

## Supplementary Material

Crystal structure: contains datablock(s) global, I. DOI: 10.1107/S1600536811049488/hb6509sup1.cif

Structure factors: contains datablock(s) I. DOI: 10.1107/S1600536811049488/hb6509Isup2.hkl

Supplementary material file. DOI: 10.1107/S1600536811049488/hb6509Isup3.cml

Additional supplementary materials:  crystallographic information; 3D view; checkCIF report

## Figures and Tables

**Table 1:** Hydrogen-bond geometry (Å, °)

*D*—H⋯*A*	*D*—H	H⋯*A*	*D*⋯*A*	*D*—H⋯*A*
C18—H18⋯O1^i^	0.93	2.58	3.377 (3)	145

Symmetry code: (i) 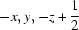.

**Fig. 1.**
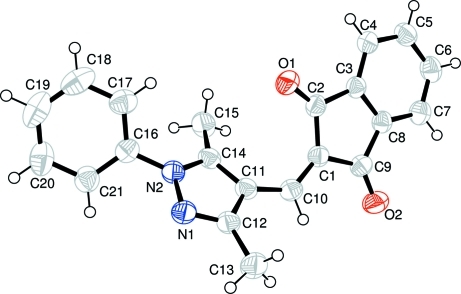
View of the title compound with displacement ellipsoids drawn at the 50% probability level. H-atoms are shown as small spheres of arbitrary radii.

**Fig. 2.**
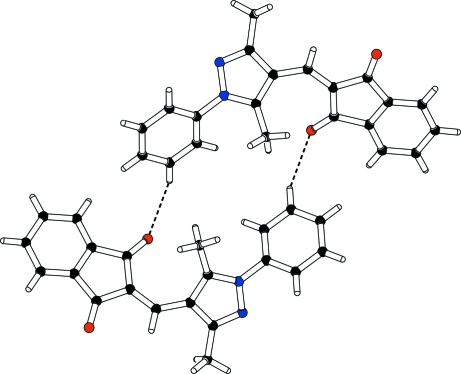
The partial packing (PLATON; Spek, 2009) which shows that molecules form dimers with R22(22) ring motif.

## supplementary crystallographic information

### Comment

Formation of the donor acceptor chromophores by the nucleophilic addition of an active hydrogen compound to a carbonyl group followed by a dehydration reaction is known as knoevenagel condensation (Asiri & Khan, 2009). Donor acceptor chromophores are applicable in the field of materials science such as third order non-linear optical (NLO) (Asiri *et al.* 2006), photonic materials and devices, optical limiting (Kulhanek *et al.* 2011), electrochemical sensing (Koyuncu *et al.* 2010) and langmuir film (Wang *et al.* 2011). Due to wide application of donor acceptor chromophores, we are reporting here the synthesiz and crystal structure of the title compound (I), (Fig. 1).

The crystal structures of (II) *i.e.*, 2-(4,5,6,7,8,9-hexahydro-6a-azaphenylen-2-ylmethylene)indan-1,3-dione (Belyakov *et al.*, 2008) and (III) *i.e.*, 4-((*E*)((3,5-dimethyl- 1-phenyl-1*H*-pyrazol-4-yl)methylene)amino)-1,5-dimethyl-2-phenyl -1,2-dihydro-3*H*-pyrazol-3-one have been published which contain the moities present in (I).

In (I), the group A (C1—C9/O1/O2) of indan-1, 3-dione, the heterocyclic five membered ring B (C11/C12/C14/N1/N2) and the benzene ring C (C16—C21) of the aldehyde moiety are planar with r. m. s. deviation of 0.0345, 0.0099 and 0.0035 Å, respectively. The dihedral angle between A/B, A/C and B/C is 39.77 (4), 21.92 (7) and 47.06 (6)°, respectively. The title compound consists of dimers due to intermolecular H-bonds of C—H···O type, where O-atom is of carbonyl and H-atom is of benzene ring. This H-bondings form a *R*_2_^2^(22) (Fig. 2) ring motif (Bernstein *et al.*, 1995). There exists π–π interactions between the centroids of the rings of indan-1, 3-dione moieties at the separation of 3.8325 (12)–3.8600 (12) A°.

### Experimental

A mixture of 3,5-dimethyl-1-phenylpyrazole-4-carbaldehyde (1.0 g, 5.0 mmol), indan-1, 3-dione (0.73 g, 5.0 mmol) and a few drops of pyridine in ethanol (15 ml) was heated for 3 h. The progress of the reaction was monitored by TLC. The solid that separated from the cooled mixture was collected and recrystallized from a methanol-chloroform mixture to give the yellow prisms of (I).

Yellow: 85%, m.p. 469–470 K.

IR (KBr) ν_max_ cm^-1^: 3035 (Ar—H), 2859 (C—H), 1663 (C═O), 1578 (C═C).

### Refinement

The H-atoms were positioned geometrically (C–H = 0.93–0.96 Å) and refined as riding with *U*_iso_(H) = x*U*_eq_(C), where x = 1.5 for methyl and x = 1.2 for aryl H-atoms.

### Crystal data

**Table:**

C_21_H_16_N_2_O_2_	*F*(000) = 1376
*M**_r_* = 328.36	*D*_x_ = 1.329 Mg m^−^^3^
Monoclinic, *C*2/*c*	Mo *K*α radiation, λ = 0.71073 Å
Hall symbol: -C 2yc	Cell parameters from 2106 reflections
*a* = 14.6655 (3) Å	θ = 1.4–25.3°
*b* = 7.8902 (2) Å	µ = 0.09 mm^−^^1^
*c* = 28.6651 (7) Å	*T* = 296 K
β = 98.251 (1)°	Prism, yellow
*V* = 3282.61 (13) Å^3^	0.26 × 0.23 × 0.21 mm
*Z* = 8	

### Data collection

**Table:**

Bruker Kappa APEXII CCD diffractometer	2970 independent reflections
Radiation source: fine-focus sealed tube	2106 reflections with *I* > 2σ(*I*)
graphite	*R*_int_ = 0.034
Detector resolution: 8.00 pixels mm^-1^	θ_max_ = 25.3°, θ_min_ = 1.4°
ω scans	*h* = −17→17
Absorption correction: multi-scan (*SADABS*; Bruker, 2005)	*k* = −6→9
*T*_min_ = 0.975, *T*_max_ = 0.985	*l* = −34→34
12302 measured reflections	

### Refinement

**Table:**

Refinement on *F*^2^	Primary atom site location: structure-invariant direct methods
Least-squares matrix: full	Secondary atom site location: difference Fourier map
*R*[*F*^2^ > 2σ(*F*^2^)] = 0.042	Hydrogen site location: inferred from neighbouring sites
*wR*(*F*^2^) = 0.112	H-atom parameters constrained
*S* = 1.01	*w* = 1/[σ^2^(*F*_o_^2^) + (0.0522*P*)^2^ + 0.9126*P*] where *P* = (*F*_o_^2^ + 2*F*_c_^2^)/3
2970 reflections	(Δ/σ)_max_ < 0.001
228 parameters	Δρ_max_ = 0.12 e Å^−^^3^
0 restraints	Δρ_min_ = −0.23 e Å^−^^3^

### Special details

**Table:**

Geometry. Bond distances, angles *etc*. have been calculated using the rounded fractional coordinates. All su's are estimated from the variances of the (full) variance-covariance matrix. The cell e.s.d.'s are taken into account in the estimation of distances, angles and torsion angles
Refinement. Refinement of *F*^2^ against ALL reflections. The weighted *R*-factor *wR* and goodness of fit *S* are based on *F*^2^, conventional *R*-factors *R* are based on *F*, with *F* set to zero for negative *F*^2^. The threshold expression of *F*^2^ > σ(*F*^2^) is used only for calculating *R*-factors(gt) *etc*. and is not relevant to the choice of reflections for refinement. *R*-factors based on *F*^2^ are statistically about twice as large as those based on *F*, and *R*- factors based on ALL data will be even larger.

### Fractional atomic coordinates and isotropic or equivalent isotropic displacement parameters (Å^2^)

**Table:**

	*x*	*y*	*z*	*U*_iso_*/*U*_eq_	
O1	0.07106 (8)	0.30881 (19)	0.11877 (4)	0.0564 (5)	
O2	0.23637 (9)	0.04176 (19)	0.00898 (4)	0.0608 (5)	
N1	0.34865 (9)	0.1867 (2)	0.23313 (5)	0.0473 (5)	
N2	0.25797 (9)	0.1475 (2)	0.23590 (5)	0.0439 (5)	
C1	0.17883 (11)	0.1490 (2)	0.07845 (6)	0.0419 (6)	
C2	0.09301 (11)	0.2414 (3)	0.08361 (6)	0.0423 (6)	
C3	0.03786 (11)	0.2486 (2)	0.03591 (6)	0.0403 (6)	
C4	−0.04905 (12)	0.3164 (3)	0.02282 (6)	0.0475 (6)	
C5	−0.08681 (13)	0.3072 (3)	−0.02408 (7)	0.0537 (7)	
C6	−0.03758 (14)	0.2345 (3)	−0.05695 (7)	0.0557 (7)	
C7	0.04935 (13)	0.1681 (3)	−0.04398 (6)	0.0519 (7)	
C8	0.08677 (11)	0.1748 (2)	0.00319 (6)	0.0414 (6)	
C9	0.17632 (12)	0.1113 (3)	0.02737 (6)	0.0450 (6)	
C10	0.25407 (11)	0.1176 (2)	0.11037 (6)	0.0441 (6)	
C11	0.26939 (11)	0.1368 (2)	0.16062 (6)	0.0418 (6)	
C12	0.35529 (11)	0.1768 (2)	0.18786 (6)	0.0441 (6)	
C13	0.44513 (12)	0.2131 (3)	0.17115 (7)	0.0616 (8)	
C14	0.20881 (11)	0.1150 (2)	0.19334 (6)	0.0418 (6)	
C15	0.11558 (11)	0.0380 (3)	0.18802 (6)	0.0559 (7)	
C16	0.22738 (12)	0.1517 (2)	0.28101 (6)	0.0440 (6)	
C17	0.14828 (13)	0.2378 (3)	0.28667 (7)	0.0577 (8)	
C18	0.12018 (16)	0.2438 (3)	0.33060 (9)	0.0709 (9)	
C19	0.17138 (19)	0.1645 (3)	0.36842 (8)	0.0743 (10)	
C20	0.25092 (17)	0.0804 (3)	0.36263 (7)	0.0682 (9)	
C21	0.27950 (13)	0.0733 (3)	0.31885 (6)	0.0536 (7)	
H4	−0.08104	0.36672	0.04493	0.0570*	
H5	−0.14557	0.34996	−0.03377	0.0644*	
H6	−0.06392	0.23049	−0.08844	0.0668*	
H7	0.08189	0.12017	−0.06624	0.0623*	
H10	0.30449	0.07647	0.09756	0.0529*	
H13A	0.49379	0.20906	0.19738	0.0924*	
H13B	0.44309	0.32379	0.15712	0.0924*	
H13C	0.45628	0.12989	0.14820	0.0924*	
H15A	0.07018	0.12581	0.18772	0.0838*	
H15B	0.11146	−0.03737	0.21392	0.0838*	
H15C	0.10477	−0.02420	0.15897	0.0838*	
H17	0.11393	0.29166	0.26111	0.0693*	
H18	0.06657	0.30145	0.33466	0.0850*	
H19	0.15205	0.16794	0.39792	0.0891*	
H20	0.28568	0.02796	0.38831	0.0818*	
H21	0.33335	0.01622	0.31488	0.0643*	

### Atomic displacement parameters (Å^2^)

**Table:**

	*U*^11^	*U*^22^	*U*^33^	*U*^12^	*U*^13^	*U*^23^
O1	0.0561 (8)	0.0723 (10)	0.0421 (7)	0.0083 (7)	0.0117 (6)	−0.0049 (7)
O2	0.0533 (8)	0.0730 (11)	0.0585 (8)	0.0118 (7)	0.0158 (6)	−0.0066 (7)
N1	0.0349 (8)	0.0581 (11)	0.0483 (9)	−0.0067 (7)	0.0035 (6)	−0.0007 (8)
N2	0.0348 (8)	0.0530 (10)	0.0433 (8)	−0.0046 (7)	0.0032 (6)	0.0014 (7)
C1	0.0384 (9)	0.0464 (11)	0.0407 (9)	−0.0030 (8)	0.0053 (7)	0.0007 (9)
C2	0.0416 (10)	0.0472 (12)	0.0390 (10)	−0.0033 (8)	0.0087 (8)	0.0025 (9)
C3	0.0412 (10)	0.0400 (11)	0.0402 (10)	−0.0041 (8)	0.0076 (7)	0.0044 (8)
C4	0.0455 (10)	0.0516 (13)	0.0465 (10)	0.0007 (9)	0.0106 (8)	0.0068 (9)
C5	0.0453 (11)	0.0605 (14)	0.0532 (12)	0.0008 (10)	−0.0001 (9)	0.0100 (10)
C6	0.0595 (12)	0.0637 (15)	0.0407 (10)	−0.0042 (11)	−0.0038 (9)	0.0023 (10)
C7	0.0575 (12)	0.0560 (14)	0.0424 (10)	−0.0013 (10)	0.0076 (9)	−0.0030 (9)
C8	0.0417 (10)	0.0424 (12)	0.0402 (10)	−0.0053 (8)	0.0062 (7)	0.0013 (8)
C9	0.0428 (10)	0.0458 (12)	0.0476 (10)	−0.0032 (9)	0.0103 (8)	−0.0001 (9)
C10	0.0392 (10)	0.0460 (12)	0.0474 (10)	−0.0028 (8)	0.0073 (8)	−0.0007 (9)
C11	0.0359 (9)	0.0448 (12)	0.0436 (10)	−0.0005 (8)	0.0020 (7)	0.0018 (8)
C12	0.0369 (9)	0.0488 (12)	0.0463 (10)	−0.0027 (8)	0.0045 (7)	0.0022 (9)
C13	0.0403 (10)	0.0857 (17)	0.0585 (12)	−0.0126 (11)	0.0060 (9)	0.0028 (11)
C14	0.0341 (9)	0.0442 (12)	0.0456 (10)	−0.0015 (8)	0.0006 (7)	0.0042 (9)
C15	0.0423 (10)	0.0678 (15)	0.0557 (11)	−0.0129 (10)	0.0009 (8)	0.0091 (11)
C16	0.0419 (10)	0.0437 (12)	0.0471 (10)	−0.0080 (9)	0.0089 (8)	−0.0040 (9)
C17	0.0499 (12)	0.0578 (14)	0.0670 (13)	−0.0009 (10)	0.0136 (10)	0.0003 (11)
C18	0.0641 (14)	0.0630 (16)	0.0935 (18)	−0.0102 (12)	0.0383 (13)	−0.0158 (14)
C19	0.1054 (19)	0.0613 (16)	0.0645 (15)	−0.0224 (14)	0.0406 (14)	−0.0123 (13)
C20	0.0982 (18)	0.0600 (16)	0.0470 (12)	−0.0066 (13)	0.0127 (11)	−0.0033 (11)
C21	0.0598 (12)	0.0527 (13)	0.0483 (11)	0.0012 (10)	0.0073 (9)	−0.0045 (10)

### Geometric parameters (Å, °)

**Table:**

O1—C2	1.223 (2)	C16—C17	1.374 (3)
O2—C9	1.220 (2)	C16—C21	1.380 (3)
N1—N2	1.3787 (19)	C17—C18	1.381 (3)
N1—C12	1.318 (2)	C18—C19	1.377 (3)
N2—C14	1.350 (2)	C19—C20	1.373 (4)
N2—C16	1.429 (2)	C20—C21	1.380 (3)
C1—C2	1.480 (2)	C4—H4	0.9300
C1—C9	1.489 (2)	C5—H5	0.9300
C1—C10	1.351 (2)	C6—H6	0.9300
C2—C3	1.487 (2)	C7—H7	0.9300
C3—C4	1.384 (2)	C10—H10	0.9300
C3—C8	1.388 (2)	C13—H13A	0.9600
C4—C5	1.381 (3)	C13—H13B	0.9600
C5—C6	1.390 (3)	C13—H13C	0.9600
C6—C7	1.379 (3)	C15—H15A	0.9600
C7—C8	1.385 (2)	C15—H15B	0.9600
C8—C9	1.482 (2)	C15—H15C	0.9600
C10—C11	1.434 (2)	C17—H17	0.9300
C11—C12	1.419 (2)	C18—H18	0.9300
C11—C14	1.392 (2)	C19—H19	0.9300
C12—C13	1.493 (2)	C20—H20	0.9300
C14—C15	1.484 (2)	C21—H21	0.9300
			
N2—N1—C12	104.58 (13)	C17—C18—C19	120.1 (2)
N1—N2—C14	112.65 (13)	C18—C19—C20	120.1 (2)
N1—N2—C16	118.54 (13)	C19—C20—C21	120.3 (2)
C14—N2—C16	128.77 (14)	C16—C21—C20	119.35 (19)
C2—C1—C9	107.17 (14)	C3—C4—H4	121.00
C2—C1—C10	130.24 (16)	C5—C4—H4	121.00
C9—C1—C10	122.11 (15)	C4—C5—H5	120.00
O1—C2—C1	128.67 (16)	C6—C5—H5	120.00
O1—C2—C3	124.66 (16)	C5—C6—H6	119.00
C1—C2—C3	106.57 (14)	C7—C6—H6	119.00
C2—C3—C4	128.51 (16)	C6—C7—H7	121.00
C2—C3—C8	109.87 (14)	C8—C7—H7	121.00
C4—C3—C8	121.62 (16)	C1—C10—H10	115.00
C3—C4—C5	117.97 (17)	C11—C10—H10	115.00
C4—C5—C6	120.46 (18)	C12—C13—H13A	109.00
C5—C6—C7	121.59 (18)	C12—C13—H13B	109.00
C6—C7—C8	118.03 (17)	C12—C13—H13C	109.00
C3—C8—C7	120.32 (16)	H13A—C13—H13B	109.00
C3—C8—C9	109.58 (15)	H13A—C13—H13C	109.00
C7—C8—C9	130.10 (16)	H13B—C13—H13C	109.00
O2—C9—C1	126.67 (16)	C14—C15—H15A	109.00
O2—C9—C8	126.62 (16)	C14—C15—H15B	109.00
C1—C9—C8	106.71 (15)	C14—C15—H15C	109.00
C1—C10—C11	130.99 (16)	H15A—C15—H15B	109.00
C10—C11—C12	125.10 (15)	H15A—C15—H15C	109.00
C10—C11—C14	129.85 (15)	H15B—C15—H15C	109.00
C12—C11—C14	105.00 (15)	C16—C17—H17	120.00
N1—C12—C11	111.71 (14)	C18—C17—H17	120.00
N1—C12—C13	119.87 (15)	C17—C18—H18	120.00
C11—C12—C13	128.37 (16)	C19—C18—H18	120.00
N2—C14—C11	106.01 (14)	C18—C19—H19	120.00
N2—C14—C15	122.38 (15)	C20—C19—H19	120.00
C11—C14—C15	130.47 (15)	C19—C20—H20	120.00
N2—C16—C17	119.90 (16)	C21—C20—H20	120.00
N2—C16—C21	119.37 (16)	C16—C21—H21	120.00
C17—C16—C21	120.70 (17)	C20—C21—H21	120.00
C16—C17—C18	119.49 (19)		
			
C12—N1—N2—C14	−0.43 (19)	C4—C3—C8—C7	0.4 (3)
C12—N1—N2—C16	−178.29 (15)	C4—C3—C8—C9	−179.10 (18)
N2—N1—C12—C11	1.86 (19)	C3—C4—C5—C6	−1.2 (3)
N2—N1—C12—C13	179.52 (16)	C4—C5—C6—C7	0.7 (4)
N1—N2—C14—C11	−1.14 (19)	C5—C6—C7—C8	0.3 (3)
N1—N2—C14—C15	167.82 (16)	C6—C7—C8—C3	−0.8 (3)
C16—N2—C14—C11	176.44 (16)	C6—C7—C8—C9	178.5 (2)
C16—N2—C14—C15	−14.6 (3)	C3—C8—C9—O2	−179.0 (2)
N1—N2—C16—C17	130.59 (19)	C3—C8—C9—C1	1.0 (2)
N1—N2—C16—C21	−47.4 (2)	C7—C8—C9—O2	1.7 (4)
C14—N2—C16—C17	−46.9 (3)	C7—C8—C9—C1	−178.41 (19)
C14—N2—C16—C21	135.2 (2)	C1—C10—C11—C12	−148.87 (18)
C9—C1—C2—O1	−173.0 (2)	C1—C10—C11—C14	34.2 (3)
C9—C1—C2—C3	3.3 (2)	C10—C11—C12—N1	179.86 (16)
C10—C1—C2—O1	−0.9 (4)	C10—C11—C12—C13	2.4 (3)
C10—C1—C2—C3	175.33 (17)	C14—C11—C12—N1	−2.57 (19)
C2—C1—C9—O2	177.3 (2)	C14—C11—C12—C13	−180.00 (19)
C2—C1—C9—C8	−2.7 (2)	C10—C11—C14—N2	179.55 (16)
C10—C1—C9—O2	4.5 (3)	C10—C11—C14—C15	11.8 (3)
C10—C1—C9—C8	−175.48 (16)	C12—C11—C14—N2	2.13 (18)
C2—C1—C10—C11	12.2 (3)	C12—C11—C14—C15	−165.60 (18)
C9—C1—C10—C11	−176.81 (18)	N2—C16—C17—C18	−178.80 (19)
O1—C2—C3—C4	−6.1 (3)	C21—C16—C17—C18	−0.9 (3)
O1—C2—C3—C8	173.65 (19)	N2—C16—C21—C20	178.66 (19)
C1—C2—C3—C4	177.46 (18)	C17—C16—C21—C20	0.7 (3)
C1—C2—C3—C8	−2.8 (2)	C16—C17—C18—C19	0.2 (3)
C2—C3—C4—C5	−179.6 (2)	C17—C18—C19—C20	0.5 (4)
C8—C3—C4—C5	0.7 (3)	C18—C19—C20—C21	−0.7 (4)
C2—C3—C8—C7	−179.42 (18)	C19—C20—C21—C16	0.1 (3)
C2—C3—C8—C9	1.1 (2)		

### Hydrogen-bond geometry (Å, °)

**Table:**

*D*—H···*A*	*D*—H	H···*A*	*D*···*A*	*D*—H···*A*
C18—H18···O1^i^	0.93	2.58	3.377 (3)	145

Symmetry codes: (i) −*x*, *y*, −*z*+1/2.
